# Supplementary figures and images for: A scenario for an evolutionary selection of ageing
Source: eLife. 2024 Nov 1;13:RP92914. doi: 10.7554/eLife.92914 (PMC11530237; doi:10.7554/eLife.92914)

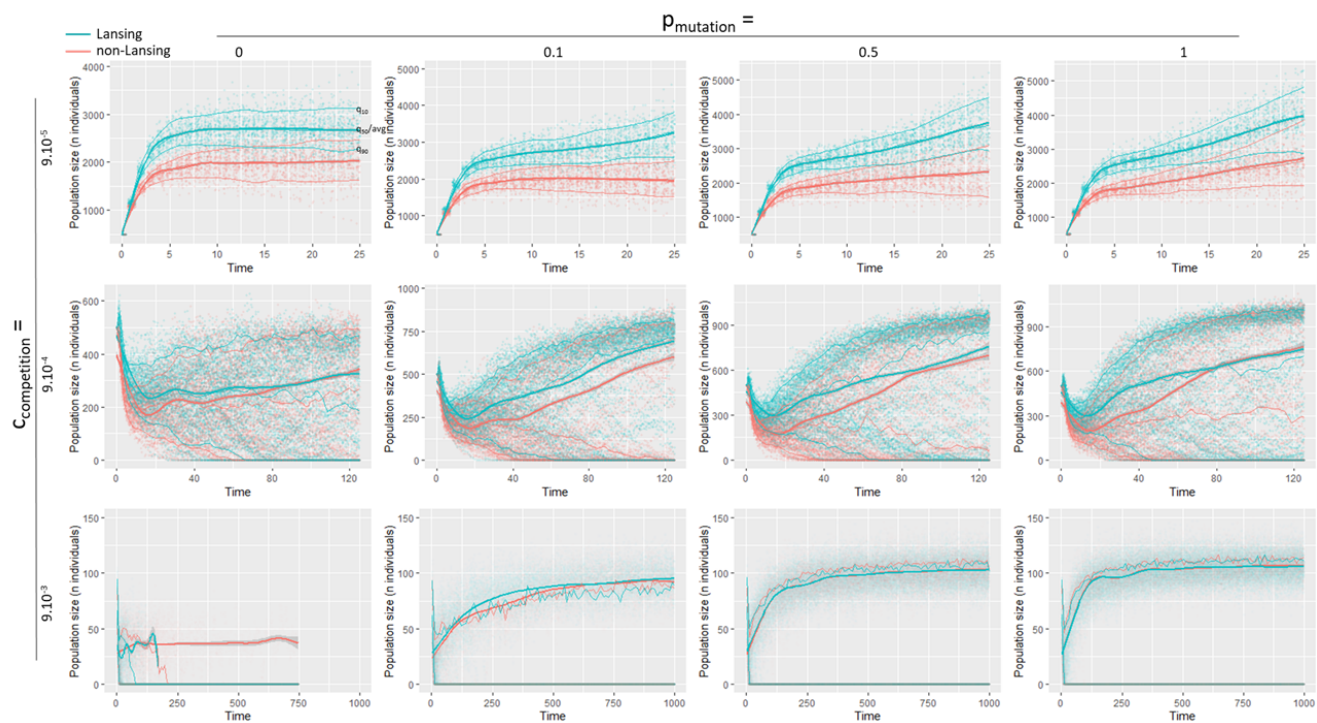

Supplement: Table 1—source data 1. — 100 independent simulations were run for each Lansing effect magnitude ranging from 0 (no Lansing effect) to 1 (progeny from parents age € [xd; xb] have xd = 0), starting with 500 Lansing (1.5; 1.3) and 500 non-Lansing (1.5; 0.83) individuals. We plot here the distribution density of xb - xd at the end of the simulation (individuals born in the time interval [990; 1000]), for Lansing populations (blue) and non-Lansing ones (red). Surprisingly, the magnitude of the Lansing effect does not seem to affect the optimal xb - xd solution value. [file elife-92914-table1-data1.zip › SuppFig2.pdf]
